# The link between gut microbiota and multiple sclerosis from the perspective of barrier function

**DOI:** 10.3389/fimmu.2025.1652796

**Published:** 2025-10-15

**Authors:** Jingru Ren, Zhenyu Niu, Jianchun Wang, Jing Guo, Hongjun Hao, Feng Gao, Ran Liu, Zhaoxia Wang

**Affiliations:** Department of Neurology, Peking University First Hospital, Beijing, China

**Keywords:** MS, gut microbiota, blood-brain barrier, brain-gut axis, leaky gut, DMTs

## Abstract

Recently, more and more studies have begun to focus on the role of gut microbiota in neurological diseases, especially immune-mediated disorders including multiple sclerosis (MS). The bidirectional communication between the gut microbiome and the central nervous system (CNS) is known as the gut-brain axis, which includes two key barriers, namely blood-brain barrier (BBB) and the gut barrier, and has become a crucial framework for understanding the pathophysiological mechanisms of various neurological disorders. Gut microbes co-evolved with humans and play important roles in maintaining steady state via various pathways, including immune regulation. An altered gut microbiota, referred to as dysbiosis, not only induces increased intestinal permeability locally, but also promotes systemic immune responses in the CNS. Increased BBB permeability has been considered the core mechanism for MS, and a “leaky” gut has also been reported in MS as well as its animal models. Therefore, the gut-brain axis is increasingly being considered as playing a crucial role in the pathogenesis of MS, with a major focus on specific gut microbiota alterations associated with the disease. Here, we review how the possible dysfunction of the gut-brain axis might impact MS, with particular emphasis on the barrier function.

## 1 Introduction

In recent years, a growing body of evidence has indicated the significant role of gut microbiota in neurological diseases, particularly multiple sclerosis (MS). The bidirectional communication between the gut microbiome and the central nervous system (CNS) is known as the gut-brain axis, a concept that has become a crucial framework for understanding the pathophysiological mechanisms of various neurological disorders. With the advancement of metagenomics and other omics technologies, the brain-gut axis is increasingly being considered as playing a central role in the pathogenesis of MS. The gut barrier and the blood-brain barrier (BBB), as two key components of the brain-gut axis, are important gateways for communication between the CNS and the gut, ensuring the selective and secure exchange of information and substances. Furthermore, increased BBB permeability has been considered the core mechanism for MS, and a “leaky” gut has also been reported in MS as well as its animal models. Studies have shown that the gut microbiota and its metabolites may play crucial roles in maintaining the integrity of these barriers via various mechanisms. And growing evidence suggests that the interaction between gut microbiota and the host immune system is also a key factor in regulating brain-gut communication. In this review, we describe the complex, multidirectional interactions between the brain and the gut microbiome in MS, mainly focusing on the impact of BBB and intestinal barrier.

## 2 Epidemiology and pathophysiology of MS

MS is a complex multifactorial disorder of the CNS that affects approximately 2 million individuals globally. It is recognized as the leading cause of neurological disability among young adults (1, 2). Characterized as a chronic neurodegenerative and neuroinflammatory condition, MS involves an aberrant immune response targeting the CNS. It is now understood that the interplay between genetic predispositions as well as environmental factors culminates in the development of demyelinating plaques in MS. Environmental risk factors include Epstein–Barr virus (EBV) infection, vitamin D3 deficiency, low UV radiation exposure, cigarette smoking, obesity, and dietary habits (3, 4). All these environmental factors would have the ability of changing the proportion of different cell subsets, leading to aberrant immune processes in the disease. The Human Leukocyte Antigen (HLA) gene complex, has been identified as a significant genetic risk factor for MS (5, 6). Therefore, among individuals with genetic predispositions, the intricate interactions between environmental triggers can surpass systemic and CNS immune tolerance mechanisms, thereby facilitating the onset of chronic inflammation and the pathogenic processes.

A recently proposed environmental risk factor for MS is the gut microbiome, a complex ecosystem comprising approximately 100 trillion microbes. Notably, the gastrointestinal (GI) tract is also recognized as the largest immune organ within the body, housing a diversity of immune cell types closely related to the gut microbiota (7). Research into autoimmune and inflammatory conditions, particularly MS and its animal model, experimental autoimmune encephalomyelitis (EAE), was among the pioneering studies in the microbiome research. Consequently, the commensal gut microbiota is now acknowledged to play a crucial role in regulating the development, homeostasis, and function of host immune systems and the CNS, particularly in MS (8, 9).

## 3 Gut microbiota and MS

### 3.1 Gut microbiota and inflammation

The human gastrointestinal tract is inhabited by a vast array of microorganisms, including viruses, bacteria, and fungi, collectively referred to as the gut microbiota (GM) (10). This microbiome establishes a symbiotic relationship with the host, wherein the host supplies nutrients and habitat necessary for microbial survival and proliferation, while the microbes contribute to the host’s health by facilitating various physiological processes (11, 12). Recent research has highlighted the capability of gut microbiome to engage in bidirectional communication with the CNS (13, 14). Consequently, the concept of the microbiota-gut-brain (MGB) axis is increasingly popular in the fields of neurobiology, medicine, and immunology.

GM significantly influences various physiological functions within the human body, including immunomodulation (15, 16). The interplay between the GM and gut immunity is pivotal in determining the occurrence and propagation of inflammation. When microbial debris and its metabolites translocate to subepithelial sites, the resultant immune response intensifies and disseminates into the systemic circulation (17). The pathogenesis of systemic inflammation associated with GM is multifaceted, and several key factors may facilitate this process, including: (1) intestinal barrier disruption, which is influenced by the equilibrium between gut mucosal immunity and luminal microorganisms (18); (2) gut dysbiosis induced by dietary habits or aging, can modify T cell activity within pro-inflammatory environment (19); (3) metabolites derived from the GM or components of gut, including small molecules and microbial components (17, 18, 20); and (4) epitope spreading and molecular mimicry mechanisms (21). Immune responses to microbial antigens may lead to tissue damage and release of self-antigens; the subsequent presentation of both microbial and self-antigens may result in the autoimmunity through a process known as epitope spreading. Molecular mimicry occurs when microbial molecules resemble host tissues.

### 3.2 Microbiota and MS

#### 3.2.1 Microbiota in MS

In fact, early evidence suggesting the involvement of the GM in autoimmune diseases can be traced back to studies on EAE. Germ-free (GF) mice, which are bred and maintained in isolators to prevent exposure to and colonization by microbiota, exhibit immunological immaturity. The mice show a marked reduction in proinflammatory Th17 cells and a skewing toward Th2 responses (22). Furthermore, GF mice demonstrate reduced severity of EAE, corresponding with lower levels of proinflammatory cytokines in the intestine and spinal cord, alongside an increase in regulatory T cells (Tregs) (23, 24). Perhaps most importantly, GM from patients with MS (PwMS), when transplanted into GF transgenic mice expressing myelin-reactive T cells, leads to an increased incidence of spontaneous EAE, which underscores the sufficiency of GM changes in MS for driving CNS autoimmunity (25).

Numerous studies have documented that PwMS experience gut dysbiosis (26–31) as summarized in **Table 1**. Specifically, PwMS exhibit an enrichment of bacterial genera such as Ruminococcus, Blautia, Dorea, Bifidobacterium, Bilophila, Sutterella, Pedobacteria, Flavobacterium, Pseudomonas, Acinetobacter, Eggerthella, and Akkermansia. Conversely, there is a reduced abundance of genera including Clostridium, Faecalibacterium, Eubacterium, Parabacteroides, Haemophilus, Adlercreutzia, Ruminococcus, Butyricimonas, Bacteroides, Coprobacillus, Lactobacillus, and Prevotella in PwMS. Furthermore, PwMS have been observed to possess a distinct mycobiome (fungus) (29, 32).

**Table 1:** Summary of MS microbiome studies.

Authors	MS type and sample size	Altered abundance of genera or OTUs in PwMS	Treatment	Geographical location	References
Cantoni C et al.	RRMS (n=24) HC (n =25)	Decreased: Prevotella, Lachnospiraceae, Anaerostipes sp., Bifidobacterium longum, Clostridium leptum, Faecalibacterium prausnitzii	All untreated	America	(27)
The iMSMS Conortium	RRMS (n=437) SPMS (n=68) PPMS (n=71) HC (n=437, paired housholds)	Decreased: Firmicutes bacterium sp., Fusicatenibacter saccharivorans, Blautia sp., Blautia obeum, Clostirum sp., Faecalibacterium praunitzii, Increased: Akkeransia muciniphila, Ruthenibacterium lactatiormans, Hungaella hathewayi, Eisenbergiela tayi	367 treated (71 Fingolimod, 86 DMF, 68 GA, 87 Interferon, 28 anti-CD20, 27 Natalizumab) 209 untreated	America, England, Spain	(28)
Thirion F et al.	RRMS (n=128) CIS (n=20) HC (n=148)	Decreased: Ruminococcus torques, Flavonifractor plautii, Lawsonibacter phoceensis, Hungatella effluvia, Pseudoflavonifractor capillosus, Erysipelatoclostridium ramosum, Ruminococcus gnavus, Sellimonas intestinalis, Coprobacillus cateniformis, and Clostridium innocuum	94 treated (32 Beta-interferon, 2 GA, 43 natalizumab, 17 fingolimod) 54 untreated	Denmark	(29)
Tremlett H et al.	Paediatric-onset RRMS (n=32) HC (n=36) ADS (n = 41)	Decreased: Anaerosporobacter sp., Ruminococcaceae, (Eubacterium) eligens, Anaerosporobacter, Clostridiales vadin BB60 Increased: Pseudomonas sp., Enterorhabdus	23 treated (11 Beta-interferon, 7 GA, 4 dimethyl fumarate, 4 others) 9 untreated	Canada, America	(31)
Jangi S et al.	RRMS (n=60) NC (n=43)	Decreased: Butyricimonas, Collinsella, Slackia Increased: Methanobrevibacter, Akkermansia	32 treated (18 Beta-interferon, 14 GA) 28 untreated	America	(34)
Takewaki D et al.	RRMS (n=62) SPMS (n=15) Atypical MS (n=21) HC (n=55) NMOSD (n=20)	Decreased: Megamonas, Roseburia,Ruminococcus sp., Eubacterium rectale, Increased: Bifidobacterium, Streptococcus, *Akkermansia muciniphila*	48 oral PSL, 12 immunosuppressive drugs, 23 Beta-interferon, 3 GA, 7 fingolimod, 1 natalilizumab, 2 DMF Untreated: not reported	Japan	(205)
Chen, J et al.	RRMS (n=31) HC (n=36)	Decreased: Parabacteroides, Erysipelotrichaceae, Lachnospiraceae, Veillonellaceae, Lactobacillus, Coprobacillus, Haemophilus Increased: Pedobacter, Flavobacterium, Blautia, Dorea, Pseudomonas, Mycoplana	20 treated (14, Beta-interferon, 1 GA, 5 Natalizumab) 11 untreated	America	(327)
Cosorich I et al.	RRMS (n=19) HC (n=17)	Microbiota isolated from the small intestinal mucosa; Decreased: Bacteroidetes, Prevotella Increased: Firmicutes, Streptococcus,	3 Fingolimod, 7 Beta-interferon, 9 GA	Italy	(338)
Cantarel BL et al.	MS (n=7) HC (N = 8)	Decreased: Bacteroidaceae, Faecalibacterium; Increased: Ruminococcus Decreased after VitD_3_: Ruminococcus, Eubacterium Increased after VitD3: Faecalibacterium, Enterobacteriaceae, Akkermansia, Janthinobacterium	5 GA with VitD_3_, 2 only use VitD_3_	America	(360)

PwMS, patients with multiple sclerosis; RRMS, relapsing-remitting multiple sclerosis; HC, healthy control; The iMSMS Conortium, the International Multiple Sclerosis Microbiome Study; SPMS, secondary progressive multiple sclerosis; PPMS, primary progressive multiple sclerosis; DMF, Dimethyl fumarate; GA, glatiramer acetate; CIS, clinically isolated syndrome; ADS, acquired demyelinating syndrome; NMOSD, neuromyelitis optica spectrum disorders; PSL, prednidolone; Rebif, an approved IFNbeta-1a formulation.

Studies also indicate the correlation between gut microbiota species richness and MS phenotypes. Patients who were clinically non-active exhibited an increased abundance of Faecalibacterium prausnitzii, Gordonibacter urolithinfaciens, Anaerostipes hadrus, Gemmiger formicilis, and Roseburia inulinivorans (28). Specific microbial taxa were also found to be linked with a reduced risk of MS relapse, such as Butyricicoccus desmolans, Odoribacter splanchnicus, Lachnospiraceae NK4A136, and Ruminococcaceae, whereas Blautia, Lachnoclostridium, Lachnospiraceae_UCG-004, and Coriobacteriales were associated with an increased risk (33). In both relapsing-remitting MS (RRMS) and progressive MS, Clostridium bolteae, Ruthenibacterium lactatiformans, and Akkermansia was observed, while a decrease in Blautia wexlerae, Dorea formicigenerans, and Erysipelotrichaceae CCM was noted. The administration of disease-modifying therapies (DMTs) also impacts the microbiota composition. For instance, reduced levels of Prevotella and Sutterella have been observed in patients with untreated MS (34).

#### 3.2.2 How microbiota influence MS

The altered gut microbiome observed in MS has fueled intense research interest in elucidating the factors that shape this microbial community and the mechanisms through which these microbes may influence MS pathogenesis. Microbiota dysbiosis can initiate a cascade of events, including the proliferation of pathogenic bacteria and the release of harmful toxins, resulting in a proinflammatory environment and a compromised gut barrier (35, 36). The leaky gut syndrome (LGS) is further characterized by increased intestinal permeability, which facilitates bacterial translocation and the growth and colonization of pathobionts, thereby triggering systemic inflammation. Numerous studies have demonstrated that EAE mice exhibit increased gut permeability (35, 37). Thus, LGS is linked to MS and can play an important role. We will discuss the specific relationship among between LGS, MS, and gut microbiota dysbiosis in **Section 3.1.3**.

Furthermore, certain bacteria can directly modulate the immune system in MS, influencing the development and behavior of immune cells, such as CD4 T cells, B cells, DCs, and macrophages, which are the main culprits in the pathophysiology of MS (38). MS is predominantly mediated by myelin-specific CD4+ T helper cells, with the Th17 cell lineage being particularly implicated. Th17 cells are known to produce the pro-inflammatory cytokine IL-17 and migrate to the CNS during active disease phase (39, 40). Concurrently, PwMS display dysregulation in CD4+ Tregs, characterized by a marked reduction in their suppressive ability (41, 42). GF mice demonstrate resistance to EAE and a lack of Th17 cells (24). However, mono-colonization with segmented filamentous bacteria (SFB) is sufficient to restore susceptibility to EAE disease and to induce the expansion of Th17 cells in the CNS (24). These findings underscore the necessity of gut bacteria in the EAE pathology. Interestingly, certain bacterial species, such as Akkermansia muciniphila and Acinetobacter calcoaceticus, are also capable of activating intestinal Th17 cells and promoting inflammation in the spinal cords of EAE mice (7, 43–45). These bacteria are found in increased abundance in the small intestine of PwMS, potentially enhancing the pathogenicity of CNS-autoreactive T cells within the intestine. The microbiota can also influence the expansion and maintenance of Tregs (32). Kasper et al. showed that the Bacteroides fragilis can enhance Treg numbers in cervical lymph nodes, which leads to amelioration of EAE (46). Moreover, alterations in gut microbiota composition may indirectly influence the capacity of Tregs to control autoimmunity by inducing of Th1 and Th17 cells or by modulating the T cell microenvironment (47). Besides well-established T cell, recent research has elucidated a gut microbiota-dependent, anti-inflammatory function of B cells in MS. Rojas et al. found a marked reduction in immunoglobulin A (IgA)+ plasma cells within the gut during EAE (48, 49). A subsequent study revealed that IgA+ B cells migrate across the BBB during active MS and exhibit specificity for MS-associated immunostimulatory bacterial strains.

Microorganisms also influence the innate immune response. Toll-like receptors (TLRs), widely distributed on immune cells and nonimmune cells including intestinal epithelial cells, neurons, and glial cells, are pattern recognition receptors (PRRs) capable of detecting exogenous and endogenous pathogenic molecules (50–52). They can be activated by microbe-related antigens like peptidoglycan, lipoteichoic acid, and LPS in the intestine, thereby initiating downstream reactions by recruiting signaling molecules through the myeloid differentiation factor 88 (MyD88) pathway or MyD88-independent signal transduction (53). MyD88 signaling results in the activation of transcription factors including the nuclear factor kB (NF-κB) and activating protein-1 (AP-1), which activate the expression of a variety of genes encoding proinflammatory cytokines and chemokines, as well as molecules important in antigen presentation. This could facilitate the reactivation of myelin-reactive T cells in the target tissue in EAE and MS (54, 55). MyD88 knockout mice are resistant to the development of active EAE, further supporting for a role of MyD88-dependent signaling in disease development (56). The composition of the intestinal flora also impacts TLRs expression, thereby influencing intestinal barrier integrity and immune homeosis (57). Microbiota is also a source of signaling molecules, immune mediators, and gut hormones, which have been shown to be involved in TLR signaling (51, 58, 59).

GMs co-evolved with humans to provide essential enzymes for digesting complex fibers, transforming humans into holobionts dependent on gut bacteria for functions such as vitamin production, nutrient digestion, and immune regulation (60–62). Dysbiosis can result in alterations in the metabolites, contributing to a proinflammatory environment (63). Recent advancements in metabolomics technology have revealed significant changes in microbial metabolites like short-chain fatty acid (SCFAs), tryptophan metabolites, bile acids, and phytoestrogens in MS. Furthermore, various classes of bacterial compounds like LPS, have been shown to penetrate systemic circulation, even reaching the CNS. We will discuss these topics in Section 3.3.

## 4 Gut microbiota and biological barrier in MS

There are two natural barriers within the BGM axis: the intestinal barrier and the BBB. Gut microbes, stress, and inflammation can alter the permeability of both structures. In this section, we will explore the roles of the two biological barriers in MS and their interactions with gut microbiota. Given the higher susceptibility of women to MS and the recent emphasis on the important role of gut microbiota in the female reproductive system and its mucosal immunity, we also briefly discuss related content.

### 4.1 Gut microbiota and BBB

BBB breakdown is an early pathological event in MS, occurring before lesion formation and in normal-appearing white matter, alongside pathogenic immune cell infiltration. Thus, limiting proinflammatory immune cells from crossing the BBB into the CNS could be an effective treatment strategy. Research indicates that gut microbiota disorders are related to BBB damage. Here, we summarize BBB components and their dysregulation in the context of gut-induced inflammation in MS.

#### 4.1.1 The composition and function of the BBB and neuron-vascular unit

The BBB refers to the barrier between plasma and brain cells, formed by brain endothelial cells (BECs), the perivascular foot processes of astrocytes, a basement membrane (BM), and pericytes (PCs). In July 2001, the National Institute of Neurological Disorders and Stroke introduced the neurovascular unit (NVU) concept to highlight the dynamic interactions between the BBB, neurons, extracellular matrix, and microglia, which collectively regulate BBB structure and function (64, 65).

BECs exhibit low penetrance to intravascular materials due to a thick luminal glycocalyx layer, specialized tight junction (TJ) structures, lack of fenestration, and selective transporters, which underpin the trans-endothelial permeability and guarantee metabolic and immunological homeostasis for normal brain functions (66). BECs also actively recruit inflammatory cells into the CNS to suppress local inflammation at BBB (67). NVU astrocytes have versatile roles, supporting vascular endothelium, responding to immune stimuli, forming endfeet and the glia limitans, and regulating intracerebral fluid flow. Astrocyte endfeet, together with secreted basal material, form the glia limitans, an immune barrier preventing T cells entry into the parenchyma (68). Connexin 43 in astrocytes helps maintain the BBB, and its loss causes continuous immune cell recruitment (69).Pericytes ensure endothelial integrity, regulating astrocytic endfeet, leukocyte trafficking, and vascular immune homeostasis and vasomotor (69, 70). Pericytes can regulate astrocytic endfeet and BBB endothelium formation. They also limit lymphocyte and monocyte transmigration into the brain (71, 72).

The BM of the BBB is a multilayered extracellular matrix composed of laminin, collagen IV, nidogen, and proteoglycan, formed by the interplay between astrocytes, BECs, and pericytes (66, 73). It allows fluid and soluble molecule passage while blocking leukocyte infiltration and binding growth factors (73). BM laminins affect T lymphocyte extravasation and migration into the brain. The perivascular space (PVS), situated between the BM secreted by BECs and astrocytes, is a key component of the highly organized glymphatic system, which include meningeal lymphatic vessels (MLVs) (69, 74). This system, characterized by astrocyte endfeet expressing polarized aquaporin-4 (AQP4) water channels, shares key functions with the peripheral lymphatic vessels and aids in CSF-interstitial fluid (ISF) exchange, waste removal, and immune cells trafficking (75, 76). The CSF exchanges with ISF through the PVS of the penetrating arteries and is ultimately drained by arachnoid granulations and MLVs, aiding nutrient delivery and metabolic waste clearance within the brain parenchyma (77, 78). Macromolecules in the subarachnoid space are transported to deep cervical lymph nodes (dCLNs) and superficial cervical lymph nodes via MLVs (79). MLVs also work with CNS immune cells, including microglial, to regulate immuno-lymphatic interface and enhance neurotrophic signaling (79, 80). Studies have shown that AQP4 serves as a crucial regulator of fluid dynamics within the brain (81).

Unlike the BECs, the choroid plexus vascular barrier (PVB) is fenestrated, allowing small molecules, water, and solutes to pass, which is crucial for CSF production (82). Animal models have demonstrated that the PVB remains permissive under normal physiological conditions but can close in response to intestinal and systemic inflammation (82, 83).

Oligodendrocytes and microglia are also crucial for BBB integrity, although they do not directly form the BBB. Seo et al. revealed oligodendrocytes can enhance TJs via TGF-β signaling (84). Studies indicate that BBB leakage initially causes astrocyte damage, followed by alterations in oligodendrocytes. Multiple mechanisms, such as imbalances in protein synthesis and degradation (85, 86), the impact of aquaporin-1 and AQP4 (87), and ionic equilibrium (88), have been suggested to explain this phenomenon.

#### 4.1.2 BBB disruption in neuroinflammation and MS

To function as an exquisite machine with highly regulatable dynamics, the BBB or NVU is crucial for brain health. Maintaining brain homeostasis requires the NVU coupling to work synergistically among BECs, pericytes, astrocytes, microglia, neurons as well as cerebral lymphatic system.

BECs, with a thick glycocalyx layer on the luminal surface as mentioned above, block macromolecule leakage and leukocyte adhesion. Hypoxia, inflammation, and TNF-α can disrupt the glycocalyx. Therefore, attenuated glycocalyx coats are involved in the early pathogenesis of neuroinflammation and brain aging. Microglia, central players in neuroinflammation, can transform into a phagocytic state and remodel neuronal connectivity, damaging the BBB by engulfing astrocyte endfeet AQP4 when peripheral inflammation breaches microvessels (89). Activated microglia also stimulate astrocytes to release TNF and glutamate (90), interacting with BECs and neurons to produce chemokines to recruit leukocytes into the CNS (91). Importantly, they also communicate with infiltrating lymphocytes and other immune cells, potentially worsening CNS inflammation (75). The cerebral lymphatic system affects MS progression by influencing immune cell movement, inflammatory responses, and oligodendrocytes function. In acute MS lesions, glial cells retraction and astrocyte damage occur, leading to reduced diffusivity along the PVS, which correlates with increased disability and longer disease duration in MS (92, 93). Impaired lymphatic fluid flow results in the accumulation of inflammatory cells and neurotoxic elements, impairing the clearance of toxic molecules and metabolites from the ventricles and deep gray matter, as well as the clearance of inflammatory microglia, thereby exacerbating cortical demyelination and gray matter pathology (94–96). MLVs facilitate meningeal T cells migration to dCLNs, and their ablation attenuates CD4^+^ T cell infiltration and spinal cord demyelination, improving EAE prognosis (97, 98). However, MLVs may also exert neuroprotective effects in MS by modulating the function of oligodendrocytes and astrocytes (99).

Chemokines, cytokines, and immune cells, may also influence BBB during systemic or local inflammation. Pro-inflammatory cytokines such as IL-1, TNF-α, and IL-6 have been linked to neuroinflammation in the CNS and peripheral nervous diseases including MS, Parkinson’s disease (PD), Alzheimer’s disease (AD) and diabetic neuropathy (100, 101). They activate signaling pathways like NF-κB and JAK/STAT, leading to neuroinflammation and BBB disruption (102–104). This disruption allows more immune cells and cytokines into the inflammation lesions, worsening inflammation in MS and other conditions (105, 106). Persistent cytokines activity leads to neuronal loss, demyelination, and chronic activation of microglia, astrocytes, and peripheral immune cells. These cytokines initiate and sustain a neuroinflammatory feedback loop, culminating in BBB breakdown, increased oxidative stress, synaptic dysfunction, and neuronal death. The persistence of this inflammatory environment is a significant element in the genesis and progression of neurological disorders, including MS (107–109). Many other cytokines like IL-22, and IFN-γ also damage the BBB by modulating TJs and increasing the expression of transmigratory molecules expression on BECs (110–112). Circulating cytokines also enhance inflammasome activation like NLRP3, which could downregulate TJ proteins and increase BBB permeability (113).

Chemokines are crucial for BBB integrity, lymphocyte chemotaxis, CNS immunosurveillance, and neural regulation (114). Studies have found the chemokines levels, such as CXCL13, CXCL9 and CCL2, are significantly increased in MS (115, 116), potentially activating the p38 mitogen-activated protein kinase (MAPK) pathway and compromising the BBB (117). The NVU controls peripheral leukocytes entry into the CNS, a process that can be disrupted by cytokines. IL-17, in particular, impairs NVU function by attracting circulating neutrophils and downregulating TJs like occludin and ZO-1 (118). Human BECs express low levels of IL-17R normally but increase expression near active MS lesions. It also facilitates CD4+T cell transmigration and enhances the ICAM-1-dependent monocyte adhesion through the BBB (119).

MS has long been seen as a T-cell-mediated disease, especially involving CD4 myelin-reactive T cells including Th1 cells and Th17 cells. Th1 cells primarily secret IFN-γ and TNF-α, which play a crucial role in activating local glial cells and antigen-presenting cells (APCs). Th17 cells can secrete matrix metalloproteinases 3 (MMP-3) and MMP-9, which can degrade the BM and facilitate peripheral leukocyte migration through the BBB (111, 120). Furthermore, Th17 lymphocytes highly express granzyme B, which subsequently kills neurons and recruits more CD4+ lymphocytes (119). Activated Th cells interact with various autoantigens like CNS resident cells, leading to a rapid clonal expansion and an amplified immune response. This ultimately triggers a cascade of inflammatory, demyelinating, and neurodegenerative events, thereby further affecting BBB permeability in MS (121). Recent studies have demonstrated that plasma cells originating from the gut, which secrete IgA, play a role in mitigating neuroinflammation in the CNS through the production of IL-10 (49). In contrast, the accumulation of IgA-producing cells that are reactive to gut bacterial strains associated with MS has been correlated with acute inflammatory episodes in MS (122).

NVU coupling relies heavily on the canonical Wnt/β-catenin, Sonic Hedgehog (SHH), PDGF-β, and TGF-β signaling pathways (123, 124). Additionally, inflammatory mediators, including mitochondrial reactive oxygen species (ROS), can stimulate proinflammatory signaling pathways (Jak-STAT, NF-κB, and NLRs) in BECs, pericytes, and astrocytes, potentially damaging the BBB and interfering with the morphogen signaling (Wnt/β-catenin and SHH) as well as transcriptional program of the BECs, leading to NVU breakage.

The BBB disruption will finally leads to transcytosis, cerebral ion metabolism imbalance, brain perfusion abnormalities, and influx of erythrocytes, cytotoxic iron, as well as fibrinogen, thrombin, and immunoglobulins, which might further drive pathology of MS. The plasminogen cascade activation is associated with MMP activity and BBB disruption in acute MS lesions (125, 126). In progressive MS, postmortem brain tissue shows increased fibrin and fibrinogen deposition in the motor cortex (127). Plasma-derived extracellular vesicles in RRMS are also enriched in fibrinogen (128). Studies reported that fibrin and fibrinogen deposition likely follow BBB breakdown, with blood-derived thrombin mediating further BBB breakdown, eliciting a Ca2+ influx, nitric oxide and ROS production, stress fibers formation, and TJ disruption in MS (129). In the NVU, BECs regulate ion transport through modulating TJ, receptors, and ion channel expression (130). In MS, the BBB disruption might impair selective ion exchange and lead to the neurotoxicity. Iron buildup has been associated with increased ROS, lipid peroxidation, decreased antioxidants, and neurodegeneration in these patients (126). Lastly, modern approaches to BBB disruption in MS also focus on the vascular changes at the NVU, where BBB function and cerebral perfusion are closely interconnected. The NVU harmoniously couples cerebral blood flow with neural activity in different regions of the brain through vascular activity, which has been reported to play a central role in MS pathology (125, 130, 131). Under MS pathology, increased NVU permeability is secondary to BECs dysfunction. Additionally, pericytes contract and undergo apoptosis, leading to capillary constriction and increased BBB damage. Global hypoperfusion in both the white and gray matter is associated with active MS with cognitive dysfunction (132). Therefore, the involvement of the NVU in MS highlights the importance of cerebral hypoperfusion in MS pathology and could represent a potential treatment target.

#### 4.1.3 The impact of gut microbiota on the BBB

Studies increasingly show that gut microbes significantly impact BBB integrity. In 2014, Braniste et al. found that GF mice exhibited higher BBB permeability in various brain regions compared to pathogen-free (PF) mice, which was linked to reduced occludin and claudin-5 expression. After fecal transplantation from PF mice or administration of SCFA-producing bacteria to GF mice restored BBB integrity by increasing TJ expression. Current research suggests that the gut microbiota regulate the BBB through a variety of pathways, including the vagus and sympathetic nerves (133), the immune system (134), the endocrine systems (135), and microbial metabolites such as SCFAs, microbial structural components such as LPS and peptidoglycans (8), and microbial membrane vesicles (136). We will discuss this part in the following section.

##### 4.1.3.1 Microbial metabolites as signaling molecules

The GM converts dietary components into various metabolites, which play crucial roles in metabolism and signaling functions, affecting host homeostasis, including BBB integrity and brain function. Recognizing the significance of structural components derived from bacterial cell walls, such as LPS and bacterial membrane vesicles, is also crucial due to their effect on host physiology. These components, often called microorganism-associated molecular patterns (MAMPs), which can play crucial roles that extend beyond innate immunity (137). In Section 4, we will discuss in detail the effects of gut microbial metabolites and their components on the biological barrier.

##### 4.1.3.2 Vagus nerve

The vagus nerve is a key channel for the communication between the intestinal microbiota and the brain. GM influence the intestinal neurons and the CNS by modifying the vagus signals to trigger anti-inflammatory reflexes, releasing mediators like acetylcholine (Ach), and interacting with immune cells (133). Vagus nerve stimulation decreases the co-localization of neutrophils and ICAM-1 induced by LPS stimulation, decreasing gene expression of hypothalamic inflammatory mediators and brain inflammatory responses (138). In a rat model of ischemic stroke, non-invasive vagus nerve stimulation was observed to reduce BBB leakage, improve TJ levels, and reduce MMP-2/9 expression, thereby protecting the BBB integrity (139).

Intestinal microbes are also bale to secrete neurotransmitters like Gamma-aminobutyric acid (GABA), 5-HT, catecholamine, and histamine (140). While being transported to the brain via circulation and neural channels, they can also activate the vagal nerve chemoreceptors by paracrine signaling, and ultimately stimulate anti-inflammatory reflexes (141, 142). They also regulate information transmission between periphery and the CNS by controlling BBB function (143, 144).

##### 4.1.3.3 Endocrine pathway

Neuroendocrine hypothalamic-pituitary-adrenal axis. The endocrine pathway allows the transfer of humoral factors to mediate bidirectional activity between the gut microbiota and the brain (145, 146). Changes in the gut microbiota structure lead to the increased intestinal barrier permeability; therefore, LPS crosses the barrier into circulation and activates the HPA axis (147, 148). As a result, mast cells are activated and corticotropin-releasing hormone (CRH) is released, resulting in increased permeability of the BBB. CRH and adrenocorticotropic hormone can also directly activate microglia to release neuroinflammatory mediators and promote the brain inflammation (149).

Enterogenous hormones. GM influence enteroendocrine cells (EECs) and production hormones, such as leptin, ghrelin, and glucagon-like peptide 1 (GLP-1), through local stimulation and production of metabolites (150, 151). GLP-1, secreted by intestinal L cells, can participate in the regulation of a variety of CNS functions including BBB integrity (152). Clostridium butyricum (Cb) boosts butyrate production in the intestinal tract, stimulating the production of gastrointestinal hormones in the colon (153). In traumatic brain injury mice, Cb supplementation reduces inflammatory reactions and intestinal permeability, thereby improving the neurological dysfunction and BBB injury, likely due to increased GLP-1 secretion (154).

##### 4.1.3.4 Immune pathway

Under normal conditions, the GM and host coexist symbiotically. When disrupted, the microorganisms and their metabolites may interact with the host immune system (155, 156). Changes in GM composition increase the intestinal permeability and trigger an immune response. Activated immune cells and the signaling molecules then reach the BBB via the blood circulation, causing systemic inflammation and elevated levels of circulating cytokines that upregulate adhesion molecules, chemokines, and MMPs in the BBB (157, 158), while downregulating TJs to increase the permeability of the BEC layer (159). The compromised BBB permits fibrin entry, which is deposited as insoluble fibrin and activates further immune response (160). Solutes and toxins entering the brain increase inflammation and attract immune cells (157), which stimulate the inflammatory signaling of the NVU (161). Thus, intracerebral inflammation and neurodegeneration are exacerbated via a vicious cycle.

As discussed above, GM can influence the BBB, brain neurons, and the endocrine and immune systems to guard against the CNS pathology associated with ageing and inflammation.

### 4.2 Gut microbiota and intestinal barrier

The human gastrointestinal tract features physical and biological barriers whose function is not only to isolate the internal host’s milieu from the outside, but also to regulate the immune system, nutrients absorption, and to limit the microorganism access. Hence, the intestinal mucosa operates in a dynamic manner to maintain intestinal integrity and immune homeostasis. Disruption in these barriers is linked not only to digestive system diseases but also to autoimmune disorders outside the gut like MS, in both experimental models and humans. In this section, we examine the multiple lines of evidence linking the intestinal barrier function and MS pathophysiology.

#### 4.2.1 The composition and function of the intestinal barrier

The intestinal barrier consists of mucus layer, epithelial barrier, and gut vascular barrier, behaving as a coordinated and multilayered network that protects host physiology from external insults and regulates several gut functions. Intestinal immune compartments are divided into inductive sites, like mesenteric lymph nodes and gut-associated lymphoid tissue (GALT), where adaptive immune cells are primed and differentiated, and effector sites, such as the intestinal lamina propria and epithelium, where these cells localize to support barrier integrity and immunity (162).

The gut epithelial barrier, made up of columnar epithelial cells (enterocytes) and specialized secretory cells (Paneth and goblet cells), is underpinned by intestinal stem cells in mucosal crypts (163, 164). The mucus layer, secreted by goblet cells, shields gut epithelial cells from harmful substances and provides a habitat for microbiota, facilitating beneficial interactions while preventing pathogen entry (165, 166). Components such as TJs, antimicrobial peptides (AMPs), secretory IgA, and glycosylated proteins contribute to this protective mechanism (167–169). Mucosal surfaces also serve as an immune barrier acting as part of the innate immune response against microbial pathogens (170). Beneath the mucus layer, the gut epithelial lining acts as a semipermeable barrier, maintaining a balance between microbial-host interactions. Enterocytes are connected by junctional complexes that regulate paracellular transport and maintain intestinal permeability (166, 171). The gut barrier also features ATP-binding cassette transporters that prevent toxin accumulation and inflammation, influenced by gut microbiota (172, 173). Enteric glial cells, similar to astrocytes in regulating BBB, can also impact gut epithelial barrier function (130, 174, 175).

The immunological layer of the intestinal barrier, following the mucus and the epithelial lining includes innate lymphoid cells and intraepithelial lymphocytes that protect against pathogens and modulate immune responses (176–178). The GALT consists of multifollicular lymphoid tissues, including Peyer’s patches, isolated lymphoid follicles, the appendix, cecal and colonic patches and rectal lymphoid tissues, with all dependent on the microbiota (179, 180). These tissues house diverse immune cells including CD4+ Th cells, Tregs, CD8+ T cytotoxic cells, DCs, macrophages, and innate lymphoid cells (ILCs) that initiate and propagate immune responses, with the IgA+ Marginal Zone B cells (MBCs) being particularly prominent. IgA+ B cells from GALT are vital for the gut-meningeal immune axis and protect the CNS from gut-derived infections (179, 181). GALT that drains mucosal surfaces will constantly encounters foreign structures from commensal microbiota, infectious pathogens and antigens, which also establish tolerance to autoantigens with the changes in autoreactive T cells phenotypes, including peptides from the CNS (182). The gut vascular barrier, with fenestrated endothelium and TJs, prevents microbial entry into circulation and controls the access of dietary compounds (183). This barrier is crucial for gut-brain axis communication, with disruptions linked to the closure of PVB in mice (83). This finding suggests a functional linkage between barriers along the BGM axis, potentially underlying the frequent comorbidity of neurological and gastrointestinal symptoms (184).

The enteric nervous system (ENS), an autonomous division of the autonomic nervous system (ANS), autonomously regulates gastrointestinal functions through its submucosal and myenteric plexuses (185). It contains neurons, glia, and immune cells, forming intrinsic circuits for gastrointestinal motility, secretion, immunity, and tissue repair (186). ENS neurons can release many neurotransmitters and are closely related to vagus efferent input, forming intrinsic sensorimotor circuits (187, 188). Enteric glial cells interact with neurons, EECs, immune cells and epithelial cells, thereby modulating barrier function (175, 189, 190). Studies of human Peyer’s patches also reveal peptidergic innervation including Substance P, Vasoactive Intestinal Peptide (VIP) and Calcitonin gene-related peptide (CGRP) immunoreactivity in cells within the GALT (191).Microbiota and enterochromaffin cell (ECC)-derived 5-HT further influence glial homeostasis. Interstitial cells, including interstitial cells of Cajal (ICCs) and platelet-derived growth factor receptor alpha-positive (PDGFRα+) cells, can facilitate gut motility via electrical coupling with smooth muscle (192, 193). Macrophages, the most abundant immune cells in the GI tract, can modulate barrier homeostasis, with their activation influencing ENS integrity and being linked to microbiota dysbiosis (194–197).

#### 4.2.2 Intestinal barrier homeostasis, the microbiome and neuroinflammation

The intricate interplay between gut microorganisms and the immune system is regulated at the gut barriers through multifaceted mechanisms. Intestinal microorganisms and their metabolites impact both immune system and intestinal epithelial barriers (IEBs), while intestinal layers reciprocally shape microbial composition and immune activity. This microbiome-mediated barrier homeostasis is pivotal in regulating of neuroinflammation.

The mucus layer critically governs gut microbial community and immune interactions. Adhesion to host epithelial cells and mucus is a key property for gut bacteria colonization, which can be modulated by host-specific mucin glycosylation (198–200). Dysregulated mucin glycosylation is correlated with increased inflammation and microbial translocation by regulating mucin degradation (201–203). The transmembrane mucins also enhance the intestinal immune functions (204). The IEB impairment is a crucial mechanism for several inflammatory and immune-mediated disorders (205). As result of gut barrier imbalances, some microorganisms, bacterial products, and toxins may translocate across the epithelium uncontrollably, leading to both the local and systemic inflammation (34, 206–209). Paracellular translocation, often linked to the impairment of TJs, has been associated with direct damage to enterocytes and their supporting structures, along with significant changes in intestinal TJs gene expression and downregulation of both ZO-1 and occludin (210, 211).

The interplay between intestinal epithelial cells (IECs) and mucosal immune components, such as intraepithelial lymphocytes and lamina propria immune cells, sustains intestinal immune homeostasis. IECs detect antigens, secrete antimicrobials, and modulate immune responses, while immune cells regulate IEC-derived cytokines (212, 213). Interactions between DCs and IECs maintain anti-inflammatory environments under steady-state conditions (214). Commensals reinforce IEB integrity via TJ regulation and IECs proliferation (215). IECs-associated inflammasomes like NLRP6 are critical for mucosal homeostasis and infection defense (216). Inflammasome-deficient mice exhibit microbiota dysbiosis, amplifying inflammatory responses and IBD susceptibility (217, 218). NLRP6 deficiency also disrupts goblet cell mucus secretion and the production of epithelial IL-18 and AMPs, impairing bacterial control (219, 220), and leading to AMPs imbalance, dysbiosis, and autoimmunity.

Additionally, as previously mentioned, gut microbiota can regulate the expression and phenotype of inflammatory cells both locally and at distant sites. Studies indicate that the microbiota in the GALT remotely influences T cell development in the thymus via soluble factors (221). In the EAE model, MOG-specific T cells proliferate substantially in the GALT under SPF conditions, but less so in germ-free environments, suggesting microbiota-induced T cells stimulation (221). Recent findings emphasize the crucial role of IgA antibody-secreting cells (ASCs) in the CNS, acting as a “brain firewall” to protect the BBB and maintain intestinal homeostasis (222). In both mice and humans, meninges contain gut-derived, commensal-specific IgA ASCs, which help prevent pathogens from entering the CNS (223). Gut bacteria stimulate secretory IgA production, which compartmentalizes commensal bacteria away from the host epithelium and modulates chemotaxis and TLRs signaling (224, 225). In mice, non-invasive bacteria residing gut are coated with IgA, promoting the production of diverse, species-specific IgA in mice (226). In an adaptation to this specific microenvironment, intestinal plasma cells (PCs) might have a distinct metabolic profile. IgA ASCs can utilize diet- and gut microbiota-derived SCFAs as one carbon source to maintain metabolism (227). Inflammatory responses induced by environmental factors or intestinal dysbiosis might dramatically change oxygenation and the metabolic profile of the PC niches in the gut.

Recently identified ILCs are key regulators of intestinal immune responses and have also been implicated in CNS autoimmunity. Among ILCs, ILC3s are notable for their similarities to Th17 cells, which are crucial in CNS inflammation and can be modulated by many cues from the gut microbiota (228, 229). ILC3s are critical for the generation of the organized lymphoid tissue in the intestinal wall and regulating microbiota content and the integrity of the intestinal barrier (230, 231). Found in different GALT compartments, ILC3 interact with immune cells including Th1 cells, Th17 cells, and Tregs, efficiently controlling effector T cells and promoting a Treg balance (232–234). ILC3s produce IL-17 to attract neutrophils to the intestine during bacterial and fungal infections (235, 236), which can also induce AMPs and TJs production (237). They are also a key source of IL-22, crucial for maintaining the intestinal barrier (238). IL-22 production is stimulated by a glial-derived neurotrophic factor from enteric glial cells in response to TLR ligands (239), and is also enhanced by SCFAs that act through AhR and FFAR, respectively (240–243).

An altered microbiome also affects bacteria-associated products that influence neuroimmune responses. Besides microbial metabolites, structural components derived from bacterial cell walls and membrane vesicles also significantly impact host physiology and gut permeability, see Section 3.3.

Overall, barrier permeability is dynamic and must be carefully orchestrated and constantly adapted to maintain homeostasis, with gut microorganisms playing a major part in achieving this goal.

#### 4.2.3 The intestinal barrier in MS: consequences of a leaky gut

The topic of intestinal permeability (IP) in neuroinflammation is actively being studied, with several lines of investigation exploring the plausible relationships between gut barrier disruption and MS, as well as on translational implications based on IP.

In a study of 12 jejunal biopsies from MS patients, Lange and Shiner observed subtle histological changes, including villous atrophy and intestinal inflammatory cell infiltration (244). The latest study used the lactulose/mannitol test to evaluate intestinal permeability in MS patients and found that 73% of cases presented with abnormal permeability (245). Elevated serum zonulin levels in both RRMS and SPMS further confirm diminished intestinal barrier function in MS, as zonulin can rapidly increase both intestinal and BBB permeability *in vitro*. Similar findings were also described in the EAE model, with increased intestinal permeability, reduced submucosal thickness, and altered TJ expression in IECs, which have been associated with a mucosal imbalance between Th1/Th17 and Treg cell subsets in intestinal lamina propria, Peyer’s patches, and mesenteric lymph nodes (41, 246). They also found that treatment with probiotic Escherichia coli strain Nissle 1917 preserved TJs and decreased intestinal permeability, leading to reduced EAE severity and decreased pro-inflammatory cytokines (41).

The above studies indicate that PwMS indeed experience an alteration in the intestinal barrier due to an altered intestinal immune response and microbial dysbiosis (247). The leaky gut may be involved in the pathophysiological process of MS via the following mechanisms. Firstly, intestinal barrier dysfunction has been associated with susceptibility to systemic infections, which are common complications in MS patients (247, 248). Furthermore, the interaction between intestinal barrier and commensal microbiota could modulate the immune response pathologically, shaping the development of immune cells such as CD4+ T cells, B cells, DCs and macrophages. Additionally, changes in intestinal permeability could exacerbate neuroimmune dysregulation by allowing transmucosal passage of injurious or immunogenic antigens. Interestingly, recent work suggests a connection between the IP changes (IPC) and MS risk factors. For example, Vitamin D deficiency may reduce intestinal calcium absorption, causing gut stasis and subsequent IPC, which would allow gut microbiota to transfer more endotoxins into the blood and trigger inflammatory cytokines production within the CNS (249).

Alterations in the gut homeostasis in MS could increase translocation of bacterial and their toxic products through an impaired intestinal barrier. A recent study found higher plasma levels of endotoxin LPS in MS, linked to *in vivo* IL-6 production and *in vitro* Th17-like responses (195). In another study, investigators also found increased LPS-binding protein levels in the serum of MS patients (196). Besides LPS, MAMPs such as bacterial lipoproteins and double-stranded RNA can enter the bloodstream and modulate the immune system through TLRs, which are present in microglia and to modulate the initiation and severity of EAE models (197). Dysbiosis may alter gut bacteria metabolites, reducing health-promoting ones like SCFAs and dietary tryptophan, which may further contribute to increased gut barrier permeability and pro-inflammatory setting. Additionally, microbiota dysbiosis disrupts IgA synthesis and AMPs production, which act as anti-inflammatory mediators beyond the gut.

Increased intestinal permeability, alterations in TJs functioning, and modifications in intestinal morphology occurred along with the changes in the immune cells including T cells, IgA ASCs and ILCs, as well as gut microbiota dysbiosis in GALT, thus indicating that disruption of intestinal homeostasis was dependent on the immune response at the initiation of EAE. Thus, the combination of LPS- and MAMPs-induced inflammation, leaky gut, metabolic imbalance, and immune activation creates a perfect storm for dysregulated immune activation that can fuel chronic disease in MS.

### 4.3 Sex and microbiota-gut-reproductive tract axis in MS

Autoimmune diseases, including MS, are more common in females, who also exhibit stronger immune responses and higher relapse rates than males with RRMS, while males face a greater risk of long-term disability progression (250–252). Mechanisms involved may include gene-environment interactions or epigenetic factors. Additionally, sex chromosome as well as sex hormone effects on peripheral and the CNS autoimmunity and neurodegeneration have been shown in MS preclinical models (253, 254).

Studies have found a relationship between microbiota and sex hormones (255). The gut microbiome influences sex hormone levels through its metabolites, the immune system, chronic inflammation, and neuroendocrine axes, including the gut-brain axis. The microbiome can metabolize estrogens via β-glucuronidase, allowing estrogen to enter the bloodstream and act on its receptors, impacting reproductive health, cardiovascular risk, metabolism, bone health, and the CNS (256). GM can also impact the function of the hypothalamic-pituitary-gonadal (HPG) axis by modulating key reproductive hormones (257). Microbiota and their metabolites, like SCFA and LPS, can impact female health by colonizing the vaginal tract. SCFAs link reproductive hormone regulation with gut microbial activity via metabolic and immune mechanisms, reducing inflammation and modulating gonadotrophin-releasing hormone (GnRH) secretion. SCFAs can suppress NF-κB activity, regulate cytokine profiles, and promote regulatory Treg activity (258), which helps establish immune tolerance at the maternal–fetal interface. Gut microorganisms influence neurotransmitter production, such as serotonin and GABA, adding a layer of neuroendocrine control over fertility by affecting GnRH pulsatility and hypothalamic communication (16, 17). This links gut health to reproductive hormone regulation. Changes in cytokine levels, like IL-6 and TNF-α, can impact endometrial receptivity and ovulation, further connecting microbial balance to reproductive outcomes (259). Sex hormones and stress affect gut motility, sensitivity, and microbiota by interacting with brain-gut axis receptors in a reciprocal manner (260). This interplay leads to the concept of microbiota-gut-reproductive tract axis (261).

The gut microbiota plays a crucial role in regulating extra-intestinal mucosal and barrier homeostasis. Key bacteria, such as Bifidobacterium, Lactobacillus, and others, are common in both the gut and vaginal tract (262). The gut microbiota also influences reproductive health by maintaining intestinal barrier integrity, which, if compromised, can lead to chronic low-grade inflammation and disrupt critical reproductive processes (257). In the reproductive system, especially the vaginal tract, microbiota protect against harmful bacteria by strengthening the mucosal barrier and producing antimicrobial substances (263). Cervical mucus acts as a barrier by trapping pathogens and enabling immune responses (264). Vaginal dysbiosis bacteria can disrupt the epithelial barrier through oxidative stress and miRNA changes, leading to cell cycle arrest, apoptosis, and necrosis, while also secreting harmful metabolites that cause immune disorders and contain factors such as IgG, IgA, and lactoferrin (265). Gut dysbiosis can trigger abnormal systemic and mucosal immune responses, increasing pro-inflammatory cytokines and cytokines and impairing embryo implantation and placental development, which is linked to infertility and repeated implantation failure (266).

Sex hormones play a role in the peripheral and central immune regulation of MS. Gut microbiota regulate the appropriate effects of sex hormones through multiple mechanisms, including metabolism, chronic inflammation, and neuroendocrine functions. However, the dysregulation of gut microbiota in MS may affect this process. Additionally, microbiota are involved in maintaining local and systemic barrier homeostasis and inflammatory processes, which play an important role in maintaining reproductive health.

### 4.4 The impact of metabolites and structural components of microbiota on biological barriers

As detailed above, compounds produced by gut microbes act locally on immune, epithelial, and EECs to affect barrier integrity, systemic immune responses, and hormone secretion (8). There are obviously many thousands of different microbiota-derived molecules that could potentially circulate to reach and penetrate the CNS. The effects of structural components derived from bacterial cell walls and of bacterial membrane vesicles on host physiology are also important extend beyond innate immunity, frequently termed MAMPs as stated above. We summarize the content covered in this article in **Table 2** and further discuss it in the following chapters.

**Table 2:** Effects of gut microbiota and their metabolities on the gut barrier or BBB.

Intervention target	Gut microbiota genera	Changes observed	Targeted barrier	Rsferences
GF mice and SPF mice	Clostridium tyrobutyricum, Bacteroides thetaiotaomicron	Maternal gut microbiota can influence prenatal development of the BBB, increased occludin, and claudin-5 in GM mice, associated with an increase in histone acetylation in brain lysates	BBB	(101)
CONV-R mouse, GF mice	unfractionated microbiota from CONV-R donor, Escherichia coli, Bacteroides thetaiotaomicron	Increased SCFA concentration, decreased GLP-1 levels and Gcg levels in the colon, accelerate intestinal transit	Gut barrier	(118)
TBI mice	Clostridium butyricum	Decreased brain edema, increased expression of the occlu- din and ZO-1 proteins in brain, increased level of Occludin, decreased level of d-lactate in serum, increased colonic GLP-1 and GLP-1R in the brain	BBB and gut barrier	(122)
GF mice and SPF mice	unfractionated microbiota from SPF mice, Lactobacillus	Increased P-gp expression in the colon	Gut barrier	(139)
GF mice and WT SPF mice, transgenic mice	SFB, Akkermansia muciniphilia, Bacteroides fragilis, Clostridium spp., unfractionated microbiota from SPF mice	Reduced gut-extrinsic sympathetic neurons activity, improve gastrointestinal motility	Gut barrier	(153)
T_84_ cell, WT SPF mice, IL-10-deficient mice	Bifidobacterium infantis (BiCM)	Increased T84 cell monolayer resistance, increased MAPK phosphorylation, increased expression of claudin-4, ZO-1, and occludin, protects against IFN- and TNF- induced permeability and TJs disruption, improved intestinal function and epithelial ionic function in IL-10-deficient mice	Gut barrier	(180)
EAE mice	Escherichia coli Nissle 1917 and K12 E. coli strain MG1655	preserved intestinal barrier function, increased antimicrobial peptides Reg3γ and Reg3β, preserved level of claudin-9 and ZO-1	Gut barrier	(192)
SPF mice, GF mice, AB mice, Vx mice, App^NL‐G‐F^ mice	unfractionated microbiota from SPF mice	reduced choroid plexus barrier function in AB mice and rescued upon gut microbiota reconstitution, reduced level of TJs and increased level of CSF IgG in GF mice and AB mice, increased levels of ZO‐1 and OCLN in SCFAs -treatmented AB mice, SCFAs treatment improves BBB integrity in Vx mice, increased levels of TJs and decreased Aβ burden in SCFAs-treatmented App^NL‐G‐F^ mice	BBB	(213)
Caco-2 cell	Escherichia coli (EPEC)	EPEC infection inhibited IFN-β induction and decreased IEC barrier function	Gut barrier	(366)

BBB, blood-brain-barrier; SPF mice, specific pathogen-free mice; GF mice, germ-free mice; P-gp, P-glycoprotein; GLP-1, glucagon-like peptide-1; BiCM, Bifidobacterium infantis conditioned medium; TJs, tight junction; AB mice, SPF mice orally with broad-spectrum antibiotics; Vx mice, vagotomized mice; App^NL‐G‐F^ mice, an Alzheimer’s mouse model; CSF, cerebrospinal fluid; SCFAs, short-chain fatty acid; Aβ, β amyloid; Caco-2 cells, colon cancer cells, can differentiate to form a polarized monolayer with functional TJs; MAPK, ZO-1, zonula occludens-1; MAPK, mitogen-activated protein kinase; OCLN, occluding.

#### 4.4.1 Diet-related metabolites and microbiota in MS

Produced by microbiota fermenting dietary fiber and resistant starch in the intestines, SCFAs (acetate, propionate, and butyrate) provide energy for both the host and the gut microbiota, and can enter host circulation and cross the BBB, enabling a role in maintaining barrier integrity (60, 267–269). Lower levels of SCFAs have been observed in MS patients (270–273). Furthermore, diminished SCFAs have been correlated with increased intestinal permeability and worsening EDSS in MS (270, 271, 273). Moreover, known SCFAs-producing gut microbiota are reduced in MS, including Butyricimonas, Bacteroides, Lachnospira, and Eubacterium (272, 273). GF mice, naturally lacking SCFAs, show a compromised BBB, while introducing butyrate or butyrate-producing bacteria like Clostridium tyrobutyricum can improve BBB dysfunction in these mice (141). The mechanism by which SCFAs influence barrier function is not fully understood. SCFAs bind G protein-coupled receptors (GPCRs) (141) and the free fatty acid receptors (FFAR2 or FFAR3) on intestinal epithelial cells and brain ECs, protecting the barrier from oxidative stress (274–278). Knox et al. also found that butyrate and propionate promote remodeling of actin cytoskeleton and TJs in an BBB model (279). In GF mice, SCFAs can improve barrier function and TJs expression at the choroid plexus in antibiotic-treated mice (280). SCFAs are also known to support mitochondrial function (281, 282), as they protect against mitochondrial disruption in brain endothelial cell treated with LPS (279). For intestinal homeostasis, SCFAs can mediate sodium transport, energize intestinal epithelial cells, and influence gene transcription that supports colon homeostasis by inhibiting histone deacetylase activity (283–285). SCFAs also reduce T cell proliferation and cytokine production in the gut, partly by inhibiting the activation of NF-κB pathway in immune cells and intestinal epithelial cells (286–288).

Bile acids (BAs), derived from cholesterol metabolites in the liver and modified in the gall bladder, become primary bile acids conjugated with glycine or taurine. Primary Bas, cholic acid and chenodeoxycholic acid (CDCA), can be further metabolized by gut microorganisms into secondary bile acids (2BAs), such as Deoxycholic acid (DCA), chenodeoxycholic acid (CDCA) and lithocholic acid (LCA) (289), which can enter systemic circulation and affect the CNS (290, 291). DCA and CDCA have been shown to have disruptive effects on the gut barrier (292, 293), whereas LCA seems to have a protective role (294). CDCA and DCA have also shown disruptive effects on the BBB in animal models, which may suggest common mechanisms of disruption across barriers (295). BAs can interact with many receptors such as Farnesoid X receptor (FXR), the VDR, PXR and Takeda G protein-coupled receptor 5 (TGR5), to exert various functions (296–298). Without these receptors, the intestinal barrier weakens, allowing the translocation of bacteria (299). Moreover, FXR modulates gut immune responses driven by microbes during inflammation, potentially linking them to BA metabolism dysregulation (300). Gut microbes can activate TGR5, affecting the expression of EECs involved in immune regulation (301). This, in turn, directly influences macrophage polarization and the subsequent inflammatory response. Once TGR5 is activated, BAs may suppress the production of inflammatory cytokines such as IL-1, IL-6, and TNF-α (302).

Tryptophan is acquired through digestion of dietary protein in the small intestine (303–305). This essential amino acid is crucial for protein synthesis and the production of serotonin (5-HT) and kynurenine (155, 306, 307). Studies have noted reduced levels of tryptophan and its metabolites in PwMS, also correlating with EDSS scores (308–310). Dietary tryptophan restriction in EAE models can abolish BBB disruption, leukocyte infiltration, and CNS demyelination, likely by inhibiting Th1/Th17 skewing and impairing migratory capacity (311). This effect is partially lost in GF mice, suggesting a microbiota-dependent mechanism. However, tryptophan and its metabolites can also exert protective effects, which are partially mediated by binding to the aryl hydrocarbon receptor (AhR). AhR regulates astrocyte and microglial crosstalk in the CNS, which controls inflammation and neurodegeneration (312, 313). Furthermore, tryptamine-mediated EAE suppression relies on AhR and modifies the gut microbiome composition to increase butyrate-producing microbiota (310).

Microbial fermentation can also produce compounds like methylamines, indoleacetate, phenylacetate, and phenolic compounds (314), as well as branched-chain amino acids (BCAAs) such as 2-methylbutyrate, isovalerate, and isobutyrate (314). BCAAs may play a role in autism spectrum disorder pathophysiology and barrier modulation (315). Gut microbes convert dietary methylamines dylcholine into trimethylamine (TMA), which is subsequently rapidly converted into TMA N-oxide (TMAO) in the liver and circulates systemically (314). TMAO can enhance BBB function through annexin A1 signaling (307, 311). Bacterial fermentation of dietary tyrosine and phenylalanine into p-cresol (314), whose metabolite, p-cresol glucuronide, protect human BECs line hCMEC/D3 upon LPS challenge (316). Kynurenine has been shown to protect barrier function in a colitis mouse model (317) and, along with tryptophan, crosses the BBB via the amino acid transporter SLC7A5 or L-type amino acid transporter 1, affecting neurotransmitter production (307).

In summary, the gut microbiome significantly influences how diet-related metabolites affect health and disease. A better understanding of how these diet- related metabolites alter the composition and function of gut bacteria could pave the way for improved treatments for PwMS.

#### 4.4.2 Microbial structural components and microbial membrane vesicles in MS

Recognizing the significance of structural components derived from bacterial cell walls and bacterial membrane vesicles is also crucial due to their effects on host physiology. Microbial structures, such as LPS and bacterial membrane vesicles, have previously been discussed as regulators of gut barrier function as well as BBB through various signals at the micro-gut-brain axis (193, 289, 318).

LPS, a component of Gram-negative bacteria cell wall, is recognized for its association with compromised gut barrier function and activation of immune system (194, 279, 280). Gut microbiota disorders can increase LPS release, leading to higher intestinal permeability and activation of gastrointestinal immune cells to release inflammatory cytokines (319, 320). In MS, elevated levels of LPS have been detected in the bloodstream (321). The same study also reported increased levels of LPS in the brain, spinal cord, and blood of EAE model (321). LPS activates TLR4 on microglia, leading to the release of inflammatory cytokines and chemokines (322), and promotes neuronal apoptosis and endothelial cells damage (323, 324). And Singh et al. showed that LPS also interacted with lipoteichoic acid on the cell wall of G+ bacteria, reduced mRNA levels of ZO-1, occludin, and JAMs, while increasing levels of TNF-α and IL-1β at the border of NVU. Additionally, LPS also affects adhesion proteins, membrane transporters, the basal lamina, and the extracellular matrix in the BBB (324). Therefore, LPS affects the integrity of BBB and NVU through a variety of mechanisms, providing a potential target for the treatment of related diseases.

Peptidoglycans, found in the cell walls of G+ and, to a lesser degree, G- bacteria, play key roles in host physiology. Bacterial membrane vesicles are lipid bilayer capsules released from the outer membranes of both Gram-negative and Gram-positive bacteria. They can transport and protect various cargoes, including proteins, DNA, RNA, metabolites, enzymes, peptidoglycans, polysaccharides, and toxins (325, 326). Gut microbial membrane vesicles can traverse the intestinal barrier, enter the bloodstream, and cross the BBB, constituting a key component of the BGM axis (200, 327). Notably, these vesicles influence gut barrier function by modulating mucosal innate immune cells such as macrophages and DCs (328). Thus, LPS and other MAMPs could constitute another pathway through which compromised barrier function impacts neuroimmune responses in MS.

### 4.5 The synergistic effect of other risk factors with microbiota dysbiosis in MS

Recent studies highlight the crucial role of the immune system’s interaction with gut microbiota as a link through which environmental factors such as Vitamin D deficiency, EBV, smoking, and obesity impact MS (83).These factors commonly disrupt immune regulation and gut microbiota, promoting MS development. This underscores the need to view MS through a comprehensive lens that considers both individual risk factors and its underlying pathogenic processes.

EBV infects over 90% of the global population and is linked to a 2–3 fold higher risk of MS after infectious mononucleosis (IM) (329, 330). MS patients show elevated EBV-specific immune responses correlate with disease activity (331–333). EBV interacts with the main genetic risk factor for MS, HLA-DRB1*1501, leading to higher Epstein-Barr Nuclear Antigen 1 (EBNA1)-specific antibody levels in carriers (334). Molecular mimicry is a key mechanism in EBV-MS immune response, with EBV proteins BamHI Rightward Reading Frame 2 (BRRF2), BamHI Fragment Rightward Open Reading Frame 3 (BFRF3), and EBNA1 exhibiting cross-reactivity with CNS autoantigens like myelin basic protein (MBP) and glial cell adhesion protein (GlialCAM) (335–337). This cross-reactive contributes to the formation of oligoclonal bands, produced by clonal B cell-derived plasma cells in the CNS (335, 338). Some findings locate this B cell response for the cross-reactive within the GALT. EBV infection induces the expression of the integrins α4β7 and CX3CR on memory B cells, which subsequently migrate to GALT, interact with the microbiota, and engage with CD4+ T cells (339). CXCR3+EBV-infected memory B cells may reactive viral antigen specific and autoimmune T cell responses in intestinal and CNS lymphoid tissues including meninges and brain parenchyma during MS, potentially stimulating CD8+ T cells and contributing to CNS inflammation (340–342). In gut lymphoid tissues, microbiota composition influences autoimmune T cell and B cell stimulation through cross-reactivity with bacteria, EBV and autoantigens (343). EBV infection also generates a large pool of antigen-presenting B cells, with latent EBV infection transforming B cells into potent antigen-presenters and inducing mutations in B cell receptors (BCRs) and co-stimulatory molecules, facilitating antigen uptake and presentation to CNS specific CD4+ T cells (344–346). EBV can infect human intestinal epithelial cells via cell contact, establishing latent infections (347, 348). This triggers immune responses that activate inflammatory pathways like NF-ĸB pathways, potential damaging the normal intestinal immune environment (349, 350). EBV latency type I genes, such as EBNA1 and LMP2A, downregulate the miR-200 family and reduce E-cadherin expression, compromising epithelial tissues integrity (351). In intestinal inflammatory diseases and gastrointestinal tumors, microbiota, especially H. pylori and its interaction with the EBV are significant. EBV latent proteins and the H.pylori Cytotoxin-Associated Gene A (CagA) synergistically enhance inflammatory signaling and oncogenic pathways, such as NF-kB and MAPKs, potentially causing gastric epithelium transformation and increased pro-inflammatory cytokines (352, 353). There has been reported that H. pylori infection is more frequent in MS, with recent data indicating its immunomodulatory proteins in MS experimental model (354, 355), suggesting a possible role of H. pylori in the disease. Colonization by H. pylori and/or EBV is linked with extra-gastric diseases and neuroinflammatory pathways, potentially affecting the gut–brain axis and leading to neurological disorders (356). Lastly, studies described the effect of virus infection, such as HIV and SARS-CoV-2, can alter the composition of the gut microbiome and metabolites (357–360). Therefore, EBV may be involved in the brain-gut-microbiota axis communication in MS through various mechanisms, including affecting gut microbiota composition and metabolites, inducing an inflammatory microenvironment in the gut, damaging the intestinal barrier and influencing the phenotypes of immune cells in the gut and CNS by cooperating and antagonizing with the bacteria.

Low VitD levels, along with insufficient ultraviolet B (UVB) exposure, increase the risk of MS. VitD acts as a steroid hormone, crucial for calcium and phosphate metabolism, immune balance, and brain function (361). Different studies demonstrated a decrease of around 41% in MS risk with increased serum Vit D level (362). VitD receptor elements (VDREs), regulated by VitD, are present in more than 80% of MS-associated genes (363). Moreover, VDR and CYP27B1, are found in the neurons and astrocytes, suggesting these cells might be involved in Vit D regulation (364). VitD also regulates immune cell epigenetics, promoting immunological tolerance in T cells, and reducing the inflammatory response, both of which contribute to MS pathogenesis (361). It also helps protect against CNS inflammation by regulating microglial and astrocytic activation and maintaining BBB integrity by reducing endothelial cell apoptosis and inhibiting TJ loss (365–367). Interestingly, reduced serum levels of EBNA-1 antibodies have been reported in vitamin D-supplemented MS patients (368, 369). VitD and its receptor help maintain intestinal balance by boosting bacterial diversity, reducing inflammation, and improving barrier function (370). Vitamin D3 can positively influence microbiota, fostering the growth of microorganisms that produce anti-inflammatory compounds beneficial to overall health (371). Vitamin D3 administration in MS increased the prevalence of the mucosal-integrity-promoting species such as Akkermansia, together with Fecalibacterium and Coprococcus (372). Studies conducted on mice found that the number of Bacteroidetes was higher in groups with VDR gene deletions or those on a low VitD diet (373). Meanwhile, microbiota-derived metabolites may modulate immune cell activity, enhancing vitamin D-mediated anti-inflammatory effects (371, 374, 375). GF mice exhibited hypocalcemia and decreased levels of 1,25-dihydroxyvitamin D and 24,25-dihydroxyvitamin D, in contrast with conventional mice, which showed elevated levels of FGF-23, an essential regulator of VitD metabolism (144). The gut microbiota may hinder the vitamin’s activity through secondary bile acids, particularly lithocholic acid, which interferes with vitamin D binding to and stimulating the VDR. Metabolic byproducts of bacteria, particularly SCFA-like butyrate, enhance intestinal expression of VDR by mitigating inflammation (376). The connection between the immune system and microbiome is clear, with vitamin D as a crucial intermediary. The interactions between vitamin D, the gut microbiota, and the immune system may also be among the determining mechanisms in the pathogenesis of MS.

## 5 Microbiota-brain-gut axis

**Figure 1:**
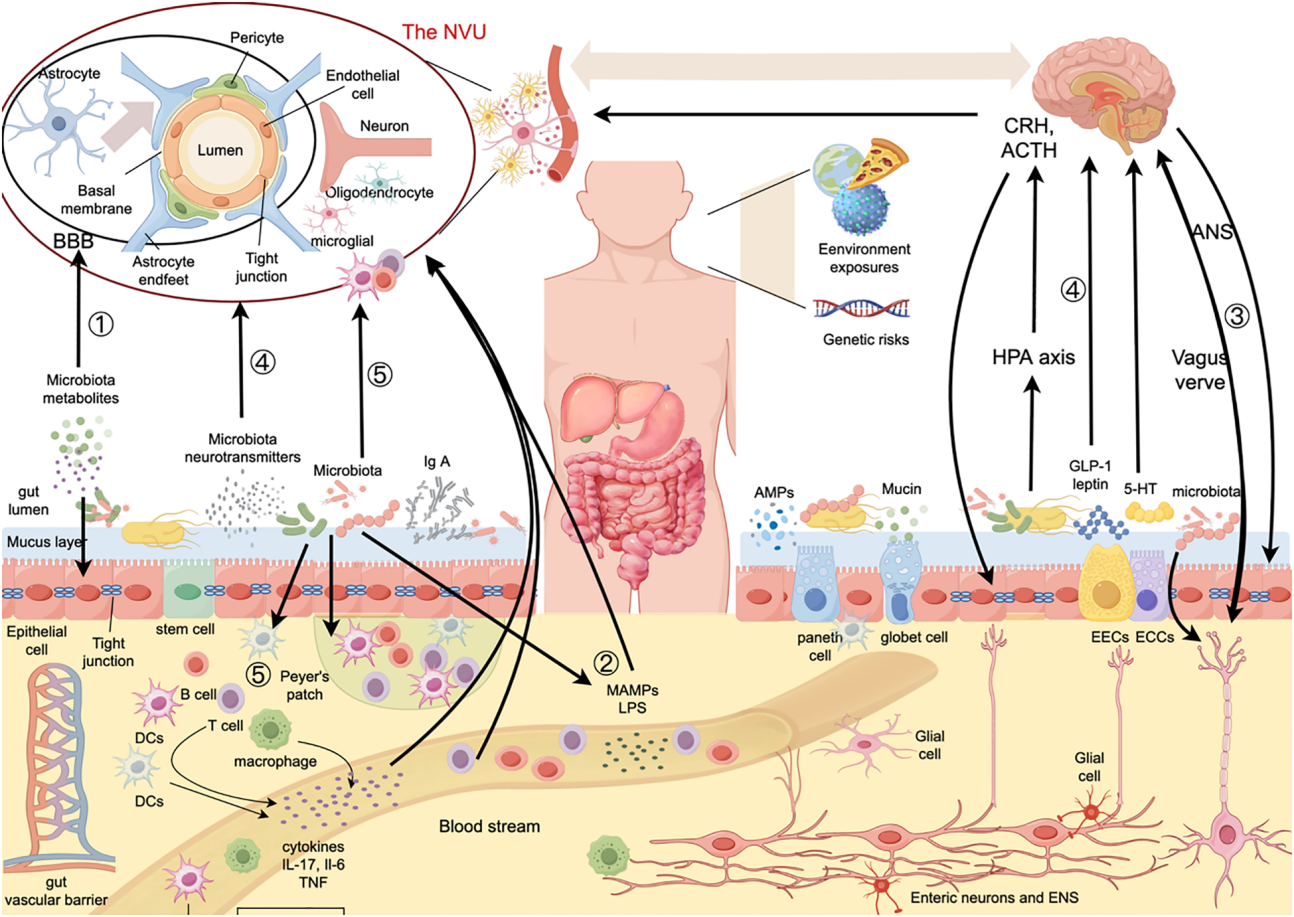
Pathways of the effects of gut microbiota on the BBB and intestinal barrier in MS. The gut microbiota can affect the structure and function of the BBB and gut barrier as well as BGM axis communications through various pathways, such as (1) microbial metabolites, which act on both the BBB and the intestinal barrier, (2) Intestinal microbiota structures, such as LPS and microbial membrane vesicles, enter the bloodstream through a “leaky gut”, act on the BBB, and enter the CNS, causing immune dysregulation, (3) The ANS regulate gut functions and influence microbiota composition and activity, among which vagal fibers can also activate enteric neurons. Additionally, the vagus nerve can sense signals from gut microbiota, enteric neurons, hormones, and peptides and transmit them to the CNS, (4) Neuroendocrine system can directly interact with microbiota via release of signaling molecules, like GLP-1, 5-HT, dynorphin, from neurons and ECCs. HPA axis, the main humoral component of the gut-brain axis, can modulate micoribiota composition and gut function by releasing glucocorticoids. The gut microbiota can also secrete neurotransmitters, such as GABA and 5-HT, which can further modulate CNS activity and the HPA axis. and (5) Gut microbiota can directly and indirectly influence immune cells of the CNS through a variety of pathways. The lymphoid tissues in the gut regulate immune cells within the gut and in the systemic circulation in conjunction with the gut microbiota. BBB, blood-brain-barrier; NVU, neurovascular unit; IgA, immunoglobulin A; MAMPs, microbe-associated molecular patterns; LPS, lipopolysaccharide; AMPs, antimicrobial peptides; HPA axis, hypothalamic-pituitary-adrenal axis; CRH, corticotropin-releasing hormone; ACTH, adrenocorticotropic hormone; ANS, autonomic nervous system; GLP-1, glucagon-like peptide-1; EECs, enterochromaffin cells; ECCs, enteroendocrine cells; ENS, enteric nervous system; 5-HT, 5-hydroxytryptamine; DCs, dendritic cells; IL-17, interleukin-17; IL-6, interleukin-6; TNF, tumor necrosis factor.

The BGM system describes the complex, bidirectional interactions between the brain, the gut connectome, the gut-associated immune system, and the gut microbiome (377). This system involves intricate signaling pathways, including neuronal (378), hormonal (379), immune (380), and microbial factors (381) to maintain homeostasis and influence various physiological processes. Alterations in these interactions are implicated involved not only in the classic functional gastrointestinal disorders, but also in a growing list of psychiatric and neurologic pathologies including MS (382–386). Here, we discuss the mechanisms of BGM axis and its role in MS, as shown in **Figure 1**.

Current evidence indicates that bottom-up modulation of the CNS by the microbiome occurs primarily through neuroimmune and neuroendocrine pathways, often involving the vagus nerve (153, 387, 388). This communication is mediated by several microbially derived molecules, including SCFAs (388–392), 2BAs and tryptophan metabolites (392, 393), which not only enter the systemic circulation but also interact with gut EECs, ECCs and the mucosal immune system locally (394–396). The microbiota can also independently produce various neuroactive molecules, such as GABA (397), 5-HT (398), norepinephrine (398, 399), and dopamine (398, 399). On the other hand, the CNS exerts regulatory control over intestinal microorganisms through multiple mechanisms, including the ANS efferent pathways and transmitter release. Furthermore, signals originating from the CNS can directly influence intestinal motility, intestinal barrier integrity, intestinal cell functions, and the living environment of intestinal microbiota, thereby impacting overall intestinal health and function.

### 5.1 Signaling mechanisms from the gut microbiota to the brain

SCFAs, BAs, and other metabolites have been implicated as signaling molecules mediating host-microbe communication via EECs and ECCs by acting on the corresponding receptors, which can regulate many CNS activities, including energy and glucose metabolism as well as HPA activity (400–404). 5-HT and its precursor, tryptophan, both play important roles in the BGM axis (401). 5-HT is mainly produced by the ECCs and is affected by gut microbiota for CNS synthesis, as the host is unable to produce tryptophan (405). The EAE model has identified direct neuroimmune regulatory roles for gut microbiota, as they can regulate immune cell trafficking and influence the development and function of the CNS-resident immune cells, particularly microglia (406–408). Relative to SPF mice, GF mice have compromised microglial maturation and morphology, resulting in weaker responses to pathogen exposure (408). Additionally, antibiotic treatment in SPF adult mice causes microglia to revert to an immature state, which can be normalized by recolonization with complex microbiota, indicating the necessity of microbial signaling throughout adulthood to preserve microglial maturation (408). Intestinal immune cells like IFN-producing meningeal NK cells and some IgA-secreting plasma cells, can directly influence neuroimmune responses, which are also regulated by gut microbiome (49, 409). Lastly, vagal receptors can detect regulatory gut peptides, inflammatory molecules, dietary elements, and bacterial metabolites to relay signals to the CNS via direct neural signaling (410), but there is also some evidence for direct activation of neurons by the gut microbiota. L. rhamnosus (JB-1), B. fragilis, and its isolated polysaccharide A all have been shown to activate intestinal afferent neurons ex vivo (411). Microbial metabolites are also candidates mediating direct activation of neurons, including microbially derived SCFAs.

### 5.2 Signaling from the brain to the gut microbiota

The ANS regulate gut functions including regional motility, secretion of gastric acid, mucus, bicarbonate, gut peptides, antimicrobial peptides, epithelial fluid maintenance, intestinal permeability, and mucosal immune response. These changes influence the microbial habitat, thereby modulating microbiota composition and activity. Vagal efferent fibers also influence immune responses and cytokine production, and they can also activate enteric neurons by synapsing with the ENS in the myenteric plexus (412–414). The sympathetic nervous system affects intestinal immune activity, while the HPA axis, the main humoral component of the gut-brain axis, responds to environmental stress or intestinal inflammation by releasing glucocorticoids and then restoring homeostasis or causing GI dysfunction by modulating enteric immune cells, gut function, and microbial composition (415).

MS can cause a variety of GI symptoms, including constipation and gastroparesis (416–419). GI function tests can also show delayed colonic transit time in MS (420). Regional intestinal transit times influence water content, nutrient availability, and microbial richness and composition (421, 422). The CNS can influence intestinal motility through multiple mechanisms, such as efferent vagus nerves, ENS, and neurotransmitters like 5-HT. Stress and inflammation can cause epithelial barrier defects by directly modulating epithelial permeability and altering the intestinal mucosal properties (420). The ANS influences mucus secretion by intestinal goblet cells, impacting intestinal mucus layer thickness and quality. Stress through catecholamine signaling can reduce mucus protective capacity and alter its composition and size (423). Changes in the intestinal barrier will further induce gut microbiota alterations.

Besides CNS-induced changes in the intestinal microbial environment, the neuroendocrine system can directly interact with microbiota via release of signaling molecules, like catecholamines, 5-HT, dynorphin, and cytokines, from neurons, immune cells, and ECCs (424, 425). Epinephrine and norepinephrine have been shown to enhance the virulence of certain enteric microbes by activating native quorum-sensing mechanisms (424, 426, 427). These findings support the notion that host neuroendocrine system can directly influence microbiota composition and function.

Therefore, pathology-associated barrier disruption may occur at several levels along the BGM axis, compromising its bidirectional communication due to the high molecular and cellular similarities. Changes in gut microbiota and microbial-derived products could contribute to damaged barriers in both gut and brain. Dysfunctional gut barriers allow these products, which could in turn reach and potentially alter brain barriers. Furthermore, gut microbiota dysbiosis could further influence barrier function by modulating neuroimmune signals. Additionally, signals from the brain, especially via the sympathetic and parasympathetic nervous systems and the ENS, can trigger intestinal inflammation and increased barrier permeability following CNS injury (428). This, in turn, will lead to gastrointestinal dysfunction, immune cell activation in the gut, gut dysbiosis, and finally escalate CNS inflammation.

### 5.3 Gut-brain communication in MS

As discussed above, gastrointestinal manifestations are common in MS. In the EAE model, gut dysbiosis causes increased intestinal permeability that precedes the CNS immune changes and induces symptoms of neuroinflammation, which suggests that gut dysbiosis promotes humoral signaling of inflammatory factors across the BGM axis (429).

Chronological age is the most significant factor influencing the clinical course of MS (430). PwMS with later onset often experience faster disability progression and poorer treatment response, possibly due to the immune system and CNS. Aging also raises the risks of age-related comorbidities like vascular and metabolic issues (431). Importantly, aging-related cognitive decline is associated with a chronic, low-grade enteric and central inflammatory state, including an increase in microglia, T cells, and border-associated macrophages in the CNS, as well as altered gut microbiota (432, 433). Aging-associated B cells also invade the meninges from the periphery and differentiate into IgM-producing plasma cells (434).

Diet-induced inflammation constitutes another important trigger in MS. It has been reported that unhealthy diet can induce cellular and neurobehavioral changes through the BGM axis (435). A Western diet, high in saturated fat and sugar, can change the expression of intestinal barrier markers, decrease EEC-derived GLP1, and induce hypothalamic inflammation through proinflammatory cytokines released by microglia (435–437). This inflammation is lessened in the absence of gut microbiome. Furthermore, transplantation of fecal content from high-fat diet–treated mice to naïve mice leads to behavioral abnormalities, indicating that diet-induced gut microbiome dysbiosis may contribute to CNS phenotypes (435). Besides diet-induced inflammation, environmental factors including obesity, viruses, smoking, tobacco use and VitD deficiency, are also considered risk factors for MS, potentially altering the microbiome and causing a leaky gut observed in PwMS and EAE animals (438).

Migration of intestinal immune cells to the CNS may also contribute to the pathogenesis of neurological and neurodegenerative diseases. In MS, the gut microbiota promotes the development of myelin-reactive Th1 and Th17 cells as well as Treg cells in the intestines, which then migrate to the CNS to promote or suppress inflammation, respectively (439). Although bacterial metabolites such as polysaccharide A and SCFAs can induce Treg cells, specific microbial components that activate proinflammatory T cells remain unclear (439).

As discussed previously, gut-derived metabolites and EECs are also crucial in the BGM axis. Metabolites from diet fibers such as SCFAs play key roles in the host’s metabolism and immune system. Metabolites like tryptophan, 2Bas have also been found to be dysregulated in both MS patients and EAE model. Changes in these gut derived metabolites and related microorganisms can exert different effects through the BGM axis. Neurotransmitters such as 5-HT, dopamine and GABA, secreted by the gut microbiome, impact the body’s defense system via immune cell receptors. A preclinical study on EAE-induced mice showed increased 5-HT levels within the neural region, enhanced 5-HT innervation of the spinal cord, and decreased EAE severity after treatment with a monoamine oxidase inhibitor (440). Therefore, the connection between these neurotransmitters and microbial regulation highlights their potent role in gut-brain interactions in neuroinflammation and MS.

In summary, the mechanism underlying the BGM communication is intricate and dynamic. Recent discoveries in the field have highlighted the significance of gut bacteria in this neuroimmunoendocrine process. This bidirectional communication not only plays a crucial role in maintaining the health of the two systems but also offers new avenues for therapy.

## 6 Therapeutic implications for barrier function in MS

Considering the impact of gut microbiota on immune modulation and barrier function, targeted interventions to normalize the gut microbiota could be a promising treatment for MS. Various approaches are currently being explored, as detailed in the following sections.

### 6.1 Probiotics

Using probiotics to replenish health-promoting gut bacteria has been suggested to maintain gut integrity and prevent pathological alterations. Probiotics are live organisms that, when administered in adequate amounts, can confer a health benefit (441). In one study, administration of probiotics increased certain taxonomic groups, such as Lactobacillus species which were depleted in MS, and decreased others that have been associated with MS, including Akkermansia and Blautia species (442). Research in laboratories and animal models has suggested multiple mechanisms by which probiotics could mediate beneficial effects, including induction of antimicrobial peptides, release of antimicrobial factors, suppression of immune cell proliferation, and enhancement of gut barrier function (443, 444). Animal models also suggest that probiotics can mitigate EAE by boosting IL-10 and TGFβ production from immune cells, amplifying Treg cell populations in gut-associated lymphoid organs and the CNS, and reducing levels of TNF, IFNγ and IL-17 (445–448).

### 6.2 Prebiotics

Prebiotics are non-digestible short-chain carbohydrates that promote some beneficial colonic bacteria production when selectively consumed (441). Their health benefits stem from their resistance to hydrolysis in the upper gastrointestinal tract and from fermentation in the large intestine by mainly anaerobic bacteria (449). Common prebiotics include disaccharides (lactulose), oligosaccharides (fructooligosaccharides, FOS; galactooligosaccharides, GOS) and polysaccharides (inulin) (450). Other sources of prebiotics include resistant starches, pectin, whole grains, and polyphenols (451). Animal studies have shown that prebiotics can influence brain function (452, 453) and modulate mood as well as stress responses by affecting the HPA axis. In healthy mice, supplementation with FOS or GOS can decrease anxiety and increase social behavior by promoting the growth of beneficial species such as Bifidobacteria (452, 454). However, human studies investigating the benefits of prebiotics on the brain are limited and inconclusive. Synbiotics, a combination of prebiotics and probiotics, have been found to improve age-related memory impairment in rats and are currently being tested in humans to promote intestinal health (455–457).

### 6.3 Fecal microbiota transplantation

FMT involves transferring fecal contents from a healthy donor into a patient, typically after broad-spectrum antibiotics, to correct disease-induced dysbiosis. It is gaining attention for treating neurodegenerative diseases by modulating gut microbiota and restoring homeostasis in the gut–brain axis primarily. Research highlights its potential in managing conditions such as PD, AD, and MS (330). In PD, FMT has been shown to alleviate motor dysfunction, reduce neuroinflammation, and regulate microbial populations by suppressing pro-inflammatory signaling pathways, including TLR4, MyD88 and NF-kB, while boosting dopamine and serotonin levels within the substantia nigra (458, 459). FMT has demonstrated benefits in reducing amyloid-β plaques, enhancing cognitive performance, and modulating neuroinflammation in AD. Furthermore, It also alters the gut metabolome, increasing SCFAs and reducing inflammatory cytokines (460). In MS, FMT has proven promising in preclinical studies by restoring gut microbial balance, reducing microglial activation, strengthening BBB integrity, and alleviating axonal damage (461). Some case–control studies have reported encouraging outcomes, showing that FMT can improve neurological symptoms in PwMS for 10 to 15 years, proving to be safe and tolerable (462, 463).

Reproducibility, scalability, and safety concerns may limit fecal FMT practice. Donor fecal material heterogeneity can cause outcome variability, and the risk of transmitting pathogens persists despite testing. A new method involves delivering defined bacterial communities instead of undefined fecal contents to colonize the recipient’s gut and restore a healthy microbiome (429). This approach is more compatible with standard manufacturing practices, addressing FMT’s limitations. Future efforts should refine FMT through personalized strategies like microbiota profiling, dietary interventions, and engineered probiotics development to enhance therapeutic results and address stability and safety issues. Understanding underlying mechanisms and advancing controlled clinical trials are critical steps toward establishing FMT as a dependable intervention for neurological conditions (330).

### 6.4 Diet and DMT

In MS, several dietary interventions have been suggested to reduce inflammation and promote clinical improvement, with some beneficial effects attributable to their impact on the gut microbiota. These interventions include the ketogenic diet, the palaeolithic diet (along with modified versions), and intermittent fasting, among others (464–467). In summary, modifying dietary patterns may be a viable, feasible, and cost-effective intervention with potential benefits in MS. Nonetheless, dietary interventions are notoriously difficult to enforce, and limited RCTs have been conducted to date. Therefore, there is a pressing need for larger, more rigorous clinical studies.

Current interventional therapies for MS, known as DMTs, include dimethyl fumarate (468), fingolimod (469), natalizumab (470), ocrelizumab (471) and others. Many DMTs have been found to alter gut microbiota composition and actively act on the barrier function, including both the BBB and intestinal barrier (372, 472–476). Glucocorticosteroids are still prescribed for acute MS relapses, which may improve BBB function by enhancing TJs and adherens junctions, and downregulate inflammation-induced endothelial CAMs *in vitro*. The first approved DMT, IFNβ, has shown stabilizing properties in biological barriers (such as the intestinal, BBB and blood–lung barriers), partly by upregulation of TJ proteins in endothelial cell layers (476). IFNβ also reduces trans-endothelial migration of proinflammatory CD4+ Th1 cells in RRMS patients (476). The commensal microbiota also can boost DCs to produce IFNβ, increasing Treg proliferation in the intestine (477). Natalizumab, a monoclonal antibody against α4β1 integrin, the cognate ligand of VCAM-1, directly disrupts the migration of immune cells, thereby reducing further damage and inflammation of the BBB. Natalizumab can also affect on integrins and lymphocyte trafficking in the gut, potentially modulating gut inflammatory in MS (477). These findings suggest that therapeutic properties of DMT in MS might rely, at least in part, on communication within the BMG axis.

As stated, probiotics and SCFAs metabolites play a colossal part in MS. Recently, the anti-inflammatory compound butyrate and its derivative, 4-phenyl butyrate, approved by the US FDA, have been shown to enhance BBB integrity and ameliorate EAE course (478). Sodium butyrate is a potent HDAC inhibitor that mainly interferes with the activity of class I HDAC enzymes. Sodium butyrate and other HDAC inhibitors, such as belinostat, exert anti-inflammatory and neuroprotective effects, which are also advantageous in acute EAE (479, 480). Besides mechanisms mediated by viable bacteria, host responses to conserved bacterial structures are crucial for intestinal homeostasis (481). Thus, immune responses can be beneficially modulated by directly sensing such bacterial structures. For instance, oral administration of commensal-derived cell wall components has been shown to promote immune tolerance in mouse models of IBD (482, 483) via specific interactions with host TLRs. These findings suggest that using either artificial ligands that mimic bacterial structures and their associated signaling responses, or isolated cell wall components from beneficial gut microbes, could support microbiome manipulation for therapy. However, care must be taken, as molecules like LPS can cross the intestinal barrier and trigger harmful immune responses. Additionally, the success of the regimen depends on delivering bacterial cell wall structures to the right place.

## 7 Conclusion

Significant advancements have been achieved over the past decade in characterizing the interaction between the gut microbiome and the CNS in various CNS inflammatory disorders. The disruption of the intricate ecosystem of the intestinal microbiota is now implicated in numerous conditions affecting both the intestine and the brain. Some pathways through which microbes influence the gut-brain axis are beginning to be elucidated. Consequently, manipulating the microbiota is garnering attention as a promising strategy for the prevention or treatment of various extra-intestinal diseases. The beneficial effects of an ‘optimized’ gut microbiota include the immune and epithelial homeostasis, enteric nervous system regulation, and optimal digestion and metabolism.

The study of the BGM system is dynamically and rapidly advancing due to ever-more-powerful biological techniques, such as metagenomics and metatranscriptomics, combined with novel bioinformatic and computational methods that enable multi-omic integration of microbial and host data via machine learning approaches. While some initial evidence suggests changes in the BGM axis in MS and EAE, the putative role of the gut microbiota and different barriers in the pathogenesis of brain autoimmunity has yet to be fully investigated. Furthermore, the causal link between an altered enteric barrier and CNS pathology has yet to be established. An appropriate test for assessing intestinal barrier impairments still needs to be established. Human studies on prodromal and untreated patients are essential to determine whether intestinal barrier changes occur early in CNS diseases or result from systemic immune and inflammatory conditions. Lastly, it is also unclear whether these intestinal barrier abnormalities are consistent across different CNS conditions. The primary trigger for the dysfunctional gut-brain axis in MS remains within a chicken-and-egg causality dilemma, with an apparent self-perpetuating loop of brain-gut inflammation.

Research on the BGM system also tends towards therapeutic transformation. Current strategies involve modifying gut microbial by altering nutrient availability to boost specific bacteria (prebiotics), introducing or expanding ‘beneficial’ species (probiotics), or transplanting entire communities or portions thereof from other intestinal donors (FMT and more selective stool transplants). Although these methods have demonstrated encouraging results in small-scale studies involving MS patients and EAE models, the absence of large-scale prospective randomized controlled trials means that these findings still suffer from limited reproducibility and challenges in generalizability across diverse populations. Furthermore, given that exogenous interventions in the gut microbiota may disrupt the intrinsic gut environment and lead to secondary complications, the aforementioned treatments possess inherent limitations. Consequently, significant progress is required before these methods can be effectively applied in the clinical management of patients with MS and other neurological diseases.

The BGM system presents several emerging research areas and unanswered questions, including the investigation of microbial metabolites and their specific effects on the host, the roles of viral and fungal components within the gut ecosystem, and the impact of environmental factors (the exposome) on the system. Further research should also focus on the role of the BGM system in different phases of the lifespan, in particular in neurodevelopmental and neurodegenerative disorders, the gut microbiota-immune system interaction, the underexplored contributions of the virome and its interactions with the gut bacteria and the interaction of the gut microbiota with the female reproductive system in maintaining homeostasis. Furthermore, integrating machine learning and artificial intelligence with multi-omics imaging and microbiome datasets has the potential to significantly enhance our understanding of the BGM system at a systems biology level. Lastly, large-scale, highly controlled, longitudinal human studies are urgently needed to identify the causes and sequelae of dysbiotic gut states and explain interindividual differences in susceptibility to BGM-related diseases.

All in all, these and other improvements in our understanding of microbe–host interactions at the gut and brain barriers will hopefully accelerate research into the use of microbiota-modulating therapies to prevent and treat neurological and neurodegenerative diseases.
